# Arbitrary numbers counter fair decisions: trails of markedness in card distribution

**DOI:** 10.3389/fpsyg.2015.00240

**Published:** 2015-03-20

**Authors:** Philipp A. Schroeder, Roland Pfister

**Affiliations:** ^1^Department of Psychiatry and Psychotherapy, Neurophysiology and Interventional Neuropsychiatry, University of Tübingen, Tübingen, Germany; ^2^Department of Psychology III, University of Würzburg, Würzburg, Germany

**Keywords:** embodied cognition, numerical cognition, SNARC effect, MARC effect, and justice for all, linguistic markedness, free choice

## Abstract

Converging evidence from controlled experiments suggests that the mere processing of a number and its attributes such as value or parity might affect free choice decisions between different actions. For example the spatial numerical associations of response codes (SNARC) effect indicates the magnitude of a digit to be associated with a spatial representation and might therefore affect spatial response choices (i.e., decisions between a “left” and a “right” option). At the same time, other (linguistic) features of a number such as parity are embedded into space and might likewise prime left or right responses through feature words [odd or even, respectively; markedness association of response codes (MARC) effect]. In this experiment we aimed at documenting such influences in a natural setting. We therefore assessed number-space and parity-space association effects by exposing participants to a fair distribution task in a card playing scenario. Participants drew cards, read out loud their number values, and announced their response choice, i.e., dealing it to a left vs. right player, indicated by Playmobil characters. Not only did participants prefer to deal more cards to the right player, the card’s digits also affected response choices and led to a slightly but systematically unfair distribution, supported by a regular SNARC effect and counteracted by a reversed MARC effect. The experiment demonstrates the impact of SNARC- and MARC-like biases in free choice behavior through verbal and visual numerical information processing even in a setting with high external validity.

## INTRODUCTION

Like nothing else, numbers are regarded as pure and objective. They are the cornerstone of scientific progress in terms of measurements and statistics and they similarly shape global business in various ways—from defining monthly salaries to describing trends at the stock market. But does this objectivity survive when numbers come in contact with human agents? In fact, there seems to be good reason for a positive answer to this question. Numbers obviously allow for rule-based decisions between competing options, and a decision that is based on numbers is readily accepted as fair and impersonal (Porter, 1996). At the same time, however, research on human decision making has documented that numbers can systematically bias an agent’s choice behavior via anchoring and adjustment heuristics (Mussweiler and Englich, 2003; Furnham and Boo, 2011). For instance, when asked to estimate the value of a property, laymen and professionals alike rated the price of a real estate higher when they were told a higher listed price before (Northcraft and Neale, 1987). This anchoring bias was found in numerous contexts and research in this domain has shown that heuristic decisions might even integrate nominally irrelevant anchors like telephone and social insurance numbers (Tversky and Kahneman, 1974).

Such anchoring effects are of course driven by memory processes rather than by the numbers themselves. Still, recent research on numerical and embodied cognition suggests that the mere presence of a number alone might be sufficient to invoke biases in thoughts and actions (Barsalou, 1999; Fischer, 2006, 2012). These biases built on well-documented associations between numerical magnitude and spatial locations that indicate smaller numbers to be associated with left locations and larger numbers to be associated with right locations [spatial numerical associations of response codes (SNARC) effect; Dehaene et al., 1993; Wood et al., 2008]. Most importantly for the present study, such spatial-numerical associations also affect response choices (Tschentscher et al., 2012; Shaki and Fischer, 2014). That is, when being confronted with smaller numbers, participants showed a preference for choosing a left vs. a right response key (Daar and Pratt, 2008) and, similarly, such small numbers involuntarily prompted left-oriented gaze directions (Ruiz Fernández et al., 2011) and small numbers were produced more likely while turning or gazing to the left (Loetscher et al., 2008, 2010). These automatic biases document that the mere presence of a number is sufficient to bias choices and behavior. Sensory and motor biases induced by the SNARC effect can be considered of high diagnostic merit for the understanding of grounded, embodied, and situated cognition (Fischer, 2012). Findings pertinent to this point range from culture-dependent finger counting habits that influence magnitude representations (Domahs et al., 2010) to bodily postures (Eerland et al., 2011) or even “unusual bodies” (Keehner and Fischer, 2012) that introduce peculiarities in spatial tasks. Together, these studies indicate that numerical associations reliably alter spatial response choices in deliberately employed highly controlled settings where the agent does not pursue any other goals except for deciding spontaneously for a spatially coded response.

**FIGURE 1:**
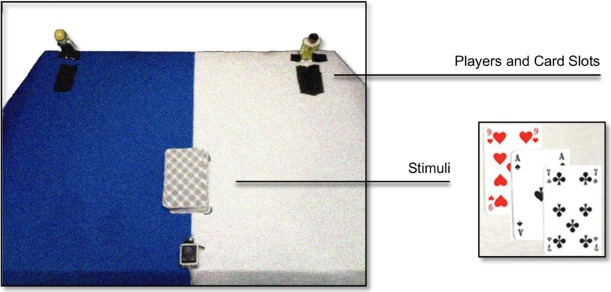
**Experimental setup.** Participants started each trial by leaving the central home key. They then drew a card, named its value and announced to assign the card to either the left or the right player (represented by two female Playmobil® characters). Card values were predefined according to the rummy game rules and explicitly instructed to the participants. A fair distribution was to be achieved without explicitly counting the values assigned to each player. The experimenter coded each announcement and we analyzed (i) how many cards and points were distributed to each player and (ii) whether digit features (magnitude and parity) affected single response choices.

As a first aim, the present study investigated whether the described bias would also occur in a more externally valid setting such as in situations where the agent aims at fairly and objectively distributing value among other people. We operationalized this situation in terms of a card distribution task in which participants were asked to deal cards of a given value to a player to the left or to the right and additionally announce their value-space choice (Figure 1). If spatial-numerical biases do indeed generalize to this situation, participants should deal more cards with higher values to the right player than to the left player.

Of course, these biases do not work in an all or none fashion, but gradually. That is, even though participants prefer choices that are congruent to a number’s spatial association (e.g., a left response to a small number), they also tend to show a fair amount of incongruent choices (e.g., a right response to a small number; Daar and Pratt, 2008). In the natural card playing setting of this study, however, both spatial-numerical associations and markedness of parity and space [markedness association of response codes (MARC) effect; Nuerk et al., 2004] might affect choice probabilities for each single card, summing up to an overall biased and therefore unfair bias in value distribution. As both, high and even numbers (such as the target card value “8” or “10”) are usually associated with right responses and with more points in the rummy card setting at hand, our main hypothesis was that participants would be biased to deal overall more points to the right than to the left player.

## MATERIALS AND METHODS

### PARTICIPANTS AND APPARATUS

Twenty-five participants (19 females, mean age = 24.3, range: 18–52 years, 3 left-handed)^1^ were invited to participate in a 15-min experimental session. They were seated in front of the apparatus displayed in Figure 1. This apparatus mainly consisted of a 60 × 40 cm cardboard box, the surface of which was covered with blue and white paper. Two Playmobil^®^ characters represented the players and were positioned at the rear edge of the card box surface with an inter-player distance of 50 cm. The players were matched for various attributes such as size, age, beauty, and orientation toward the participant. A slot in front of each player allowed the participants to insert a card in a box beneath the surface of the apparatus, restricting visual feedback of the current distribution. A central key was positioned at the front edge to allow for a standardized trial procedure, and the card deck was placed 10 cm from the key onto a predefined mark. One participant decided to distribute cards by color and thereby achieved a totally fair distribution; this participants’ data was excluded from the analysis and we refer to the remaining *N* = 24 participants in the following. The study was conducted in accordance with the Declaration of Helsinki and the guidelines of the ethics committee at the University of Würzburg.

### PROCEDURE

The basic task of the participants was to draw a card and deal it to either the left or the right player. Each participant received three random training cards, then a complete 52 Anglo-American style rummy card pack. We ensured that the card icons were printed in all four corners of each card to avoid systematic influences originating from the specific stimulus set (Figure 1). Card values were defined following standard rummy game rules, that is: number cards (2–10) counted their printed value (i.e., two points for a “2,” three points for a “3,” and so on), royal cards (jack, queen, and king) counted 10 points, and aces counted 11 points. The deck was professionally shuffled prior to the experiment. During the instructions, we emphasized that participants should aim for a fair distribution of values across players by intuition and without using any explicit strategies (such as counting points across the experiment).

To start a trial, participants pressed and released the start button. They then drew the top card from the deck, read out loud the card’s face (e.g., “Ace of Spades”), its value (“11”), and announced the side they wanted to distribute it to (always in this order). They then inserted the card into the right or left card slot. The experimenter registered the information and also coded invalid trials (i.e., illegal use of the left hand, reading out the wrong number or value, or naming the card’s attributes and the corresponding choice in the wrong order; 4.4% trials in total).

### DATA TREATMENT

For the main analysis, both the number of cards and the resulting scores for each player and participant were computed. Note that although the two measures are confounded, they still allow for distinct evaluation of choice preference and influences of the SNARC or the MARC effect: Even without an overall preference of one player in terms of the number of cards, a difference in scores can arise from a SNARC-like distribution of high-value cards to the right player and low-value cards to the left player. Both measures were controlled for homogeneity and normal distribution and subjected to one-tailed paired *t*-tests to assess our main hypothesis of a preference for the right player.

In a second, exploratory analysis, we aimed at dismantling underlying SNARC and MARC influences to the free, binary choice at a trial-wise level. Therefore, we used generalized mixed-effects models to predict the likelihood of a left response from the two first-level fixed factors parity and magnitude.

## RESULTS

### SCORES AND NUMBER OF CARDS

**FIGURE 2:**
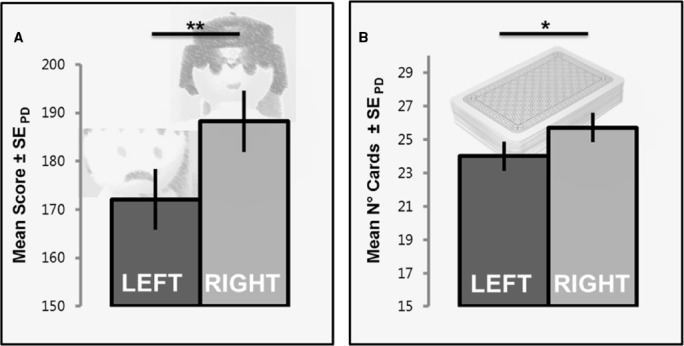
**Mean scores and standard errors of paired difference (cf. Pfister and Janczyk, 2013) in the card distribution task.** Participants overall preferred the right player which resulted in a significant difference in scores **(A)**, and a similar effect in overall card numbers **(B)**. Note that both Playmobil players acted earnest without any particular facial expression during the experiment, unlike the displayed emotions in panel **(A)**. *p < 0.05, **p < 0.01.

Mean scores and number of cards for each player are depicted in Figure 2. Tests for normal distribution (Kolmogorov Smirnov: *p*s > 0.23) and homogeneity of the sample were conducted prior to the analysis and showed the data to be suitable for analyses via parametric tests.

Whereas 188 (SE = 3.33) points on average were assigned to the right player, only 172 (SE = 3.76) points were assigned to the left player, and this difference in scores was significant, *t*(23) = 2.52, *p* = 0.010, *d* = 0.53 (Figure 2A). A similar effect emerged for the number of cards dealt to the left and right player, respectively, *t*(23) = 1.92, *p* = 0.034, *d* = 0.40 (Figure 2B), as participants assigned about two cards more ($\bar{d_{N}}$ = 1.71, SE = 0.48) to the right player. The effects on points and card numbers were correlated significantly across participants, *r* = 0.85, *p* < 0.001, indicating that the difference in cards accounted for about 71% of the effect on distributed points.

### EXPLORATORY ANALYSIS: SNARC AND MARC EFFECTS

More fine-grained analyses targeted the outcome of individual decisions rather than the overall number of points or cards dealt by each participant (Table 1). More precisely we aimed at analyzing the impact of magnitude and parity on the outcome of a decision (i.e., the likelihood for a card to be dealt to the left or to the right). To this end, we employed generalized linear mixed-effects models to model the binary outcome of the choice. Magnitude and parity were entered as fixed factors into the model (first level predictors), which further included individual subjects as random effects on the second level. The model was fitted in R by using the *glmer* function of the lme4 package (Bates et al., 2014; binomial family and logit link function). We further restricted the analysis to single-digit values (2–9) due to the actual different pictorial presentation of royal card values and possibly different representational format of values that would imply a two-digit numerical notation (Nuerk and Willmes, 2005; Nuerk et al., 2011).

**TABLE 1:** **Probabilities of left response choices as a function of target card value**.

	**Single-digit cards**								**Royal cards**	
Target card value	2	3	4	5	6	7	8	9	10	11
*P*(left) [%]	57.3	50.4	48.3	42.0	52.8	40.6	48.5	42.4	49.4	47.2

In a first step, we evaluated each predictor individually (each being coded as centered variable). As suggested by the main analyses above, higher magnitudes were indeed associated with a higher preference for right responses (fixed effect estimate = 0.055/number, SE = 0.032), *z* = 1.70, *p* = 0.045, *p_pb_* = 0.036.^2^ Surprisingly, even numbers were more likely to be dealt to the left side as compared to odd numbers (fixed effect estimate = 0.281, SE = 0.147), indicating a reliably reversed MARC effect, *z* = –1.91, *p* = 0.028, *p_pb_* = 0.030.

For model comparisons, we fitted a null model including only an intercept on the first level, an additive model with value and parity as independent predictors, and a saturated model with main effects as well as the two-way interaction. In a first step, we compared the null model to the additive model. This comparison yielded a marginally significant effect in favor of the additive model χ^2^(2) = 5.42, *p* = 0.067, *p_pb_* = 0.069, indicating that the two additional parameters did indeed add explanatory value. Further including the interaction effect, however, did not improve model fit significantly, χ^2^(1) = 0.01, *p* = 0.941, *p_pb_* = 0.929.

## DISCUSSION

We investigated the effects of different characteristics of numbers (values of playing cards) on biases in fair distribution behavior. Indeed, we found evidence for such systematic biases in a free choice experiment: Participants read out loud a rummy card’s value and announced their spatial assignment to a leftward or rightward positioned player. Without applying explicit strategies, participants failed to distribute cards in a statistically fair way and assigned a mean benefit of two cards or 16 points to the right player. In line with recent findings from the linguistic markedness and spatial-numerical associations of response codes effects, we hypothesized such a pattern to be partly driven by odd and high numbers being associated with rightward oriented action codes.

In the following exploratory analyses, we aimed at dismantling SNARC and MARC-like effects on response decisions at an individual, trial-wise level. Indeed, we found some evidence for the regular SNARC effect, but the data also indicated a reversed MARC effect with odd numbers being more likely to be distributed to the right player and even numbers being more likely to be distributed to the left player. Although this latter finding certainly comes unexpected, several recent studies cast doubt on a stable left-right association of odd and even numbers. Rather, the direction of the MARC effect seems to depend on task rules, i.e., affirmative answers seem to be generally compatible with right response codes and might override the parity-driven code of an odd number (Cho and Proctor, 2007). Further, Nuerk et al. (2005) observed the MARC effect to be altered by stimulus and experimental settings: Whereas participants showed a usual MARC effect for number words when the experiment started with Arabic notation digits, this effect was reversed when the experiment started with dice-dot patterns. In light of the apparent similarity of dice patterns and the patterns printed on the playing cards of the current experiment (see Figure 1), one might speculate that such gambling-related stimuli might generally elicit a reversed linguistic markedness of parity; however, Chang and Gibson (2011) found a regular odd-even effect in Sudoku puzzles and future studies are needed to clarify these speculations and investigate the underlying mechanisms.

Such flexibility of the MARC effect further seems likely in light of various findings on flexible coding of the related SNARC effect. For instance, the SNARC effect is influenced by inter-individual characteristics such as finger counting habits (Fischer, 2008), cultural aspects such as reading direction (Shaki et al., 2009; Domahs et al., 2010) as well as sex (Bull et al., 2013) and age (Wood et al., 2008). The MARC effect, similarly, was recently found reversed for left-handers (Huber et al., 2014), which supports a body-specificity account (Casasanto, 2009) rather than a linguistic markedness account (Nuerk et al., 2004). Furthermore, the SNARC effect is also modulated by short-term, contextual factors such as recently encountered episodes (sequence effects: Pfister et al., 2013), number usage (number placement in text: Fischer et al., 2010; on a ruler vs. clock face: Bächtold et al., 1998) and current number range (Dehaene et al., 1993; Fias et al., 1996).

### GENERAL PLACEMENT PREFERENCES

Of course, the overall preference for the right card slot of our mostly right-handed participants also reminds of robust phenomena unrelated to the processing of numerical stimuli such as turning biases when confronted with a decision to take either a left or a right turn (Liederman and Kinsbourne, 1980; Güntürkün, 2003; cf. Shaki and Fischer, 2014, for the interplay of number processing and turning during walking). Furthermore, physical positioning was shown to produce more positive attitudes for rightward placed items (Nisbett and Wilson, 1977; Choi and Myer, 2012). *Vice versa*, positive abstract concepts were associated with right space for right-handed participants (Casasanto, 2009). In fact, a vast amount of marketing literature is concerned with devaluation of laterally placed items (Dittrich and Klauer, 2012), which is at times confounded with a desirable perception of magnitude (i.e., heaviness perception; Deng and Kahn, 2009) or automatic price and quality inferences (i.e., expensive and high-quality items on the right end; Valenzuela and Raghubir, 2009). For free choice actions, goal keepers were found more likely to dive to the right during shoot-outs and under pressure (Roskes et al., 2011; but see Price and Wolfers, 2014), which was taken to document approach motivation (Roskes et al., 2014).

### HANDEDNESS-DEPENDENT PLACEMENT PREFERENCES

Already for spontaneous turning biases, stronger right-sided head-turning was documented for right-handed than for left-handed participants (Ocklenburg and Güntürkün, 2009). Similarly, positive abstract concepts were associated with rightward space for right-handers, but left-handed participants with similar linguistic experience (i.e., use of metaphors) showed a reversed association of abstract concepts and space (Casasanto, 2009), suggesting that bodily experiences might shape valence-specific placement preferences. In a large Moroccan sample that exhibited strong taboos against the use of left hands, the implicit space-valence association was found effectively identical compared to a Spain sample (de la Fuente et al., 2014), but explicit measures (i.e., good-is-right rating and ratio of right/left-handers) were larger in the Arab population. Thus, handedness and according interactions with the external world appear to be valid candidates in explaining general and explicit spatial mappings of valence.

Given the data at hand, we cannot provide evidence for culture or hand-experience specific modulations. However, valence-space and value-space associations are not necessarily interchangeable, despite a possible positive connotation of playing cards or numbers in general. For mere numbers, reversing the polarity of a response side through response eccentricity did not affect spatial-numerical associations (Santiago and Lakens, 2014), suggesting that the link between numbers and space is not (exclusively) driven by their value-valence correspondence (i.e., polarity correspondence; Proctor and Cho, 2006). Another study even suggested magnitude to underlie spatial valence representations (Holmes and Lourenco, 2011). Furthermore, number-space associations are manifold regarding the number’s features (see Patro et al., 2014, for a recent taxonomy proposal at an early age), and we next discuss the possible interpretation of SNARC and MARC effects in terms of linguistic markedness.

### LINGUISTIC MARKEDNESS IN NUMBER PROCESSING?

It is widely accepted that number processing includes a verbal component, as suggested by the triple-code model (Dehaene et al., 1993; Klein et al., 2014). Semantic features of the number (parity and magnitude) are activated automatically and can deteriorate unrelated task processing already in children of 10 years of age (Berch et al., 1999). As such, linguistic markedness of a verbal number-code, i.e., in form of the non-marked *even* parity feature, might facilitate equally non-marked responses, i.e., *right* actions (Nuerk et al., 2004). Arguably, in this experiment, the number of cards dealt to a player can be regarded an unspecific placement preference and explained a substantial proportion, but not all variance of differences in scores. Rather, the results from our exploratory analysis suggest that space-number associations further biased the distribution outcome, and that reversed space-parity associations supported but space-magnitude associations counteracted the fair distribution.

For linguistic influences in the SNARC effect, instead of assuming an oriented mental number line (i.e., Göbel et al., 2001), it is similarly possible that magnitude is coded by opposed small/large polar or linguistic representations (c.f. Nuerk et al., 2004; Proctor and Cho, 2006). Facilitated left/right responses can be accounted for by corresponding pairs of markedness: The adjectives *large* and *small* are lexical opposites with *large* as the non-marked adjective (Jakobson, 1931; see also: Lehrer, 2008). Similarly, the adjective *right* is linguistically non-marked (Zimmer, 1964), and the correspondence of both non-marked (i.e., *large* and *right*) and marked (i.e., *small* and *left*) pairs would lead to the SNARC effect. Homogenous marked and non-marked pairs should be responded to faster and they should more often be selected in a free choice paradigm. Consequently, with a decreasing marked property of *small*, the marked *left* response side was chosen less frequently. However, it is not clear how linguistic markedness can account for flexible magnitude-space and reversed parity-space associations; instead, a flexible, body-specific conceptual layer, i.e., in form of polarity or space, seems more likely. Obviously, participants were more cautious in distributing high-value (i.e., royal) cards more equally in order to distribute the cards fairly; nevertheless, magnitude-response correspondence, as indexed by the regular SNARC effect, could have effectively led to the observed right-bias.

Crucially, the interpretation of the SNARC effect in terms of polarity correspondence (Proctor and Cho, 2006) or verbal codes (Gevers et al., 2010) does not exclude the possibility of a visuo-spatial representation of magnitude. In line with the dual-coding framework of Paivio (1986), non-verbal and verbal representations can be processed referentially and activate each other. The observed SNARC effect in verbal and following motor responses can be attributed to such a referential activation. Possibly, a visuo-spatial representation was pronounced because our participants performed actual hand movements in a well-defined space, namely over a card-playing table.

We excluded two-digit and royal card stimuli from the mixed-effects SNARC and MARC models as too little is known about these indirectly magnitude-related stimuli at this time: Do they extend the mental number line similar to 0 (Pinhas and Tzelgov, 2012)? How are nominal two-digit numbers processed when part of this specific number range (Dehaene et al., 1993; Nuerk and Willmes, 2005; Nuerk et al., 2014) and does the pictorial presentation, i.e., of a king vs. a jack, trigger marked representations other than the rule-based card value?

Notwithstanding these open issues, a range of recent papers addressed the linkages of brain mechanisms devoted to language and action, respectively, and elaborated these linkages in several frameworks to accommodate for SNARC and MARC effects (e.g., Pulvermüller, 2005; Barsalou, 2008; Fischer, 2012). In case of the SNARC effect, interestingly, language or number processing is *most likely* only indirectly associated with motor system activations through magnitude processing (Fias et al., 1996) and magnitude-related spatial codes (Gevers et al., 2006) or verbal codes (Gevers et al., 2010). Still, this indirect loop was demonstrated sufficient to modulate deliberate action selection (Daar and Pratt, 2008; Ruiz Fernández et al., 2011). In this experiment, we further show that this bias even transfers to a more natural card playing scenario and is able to interfere with a fair distribution task.

### FAIR DECISIONS IN CARD DISTRIBUTION

Although statistically the goal of fair distribution was not met, participants were mostly confident about their choices during debriefing and reported to have achieved the goal by deciding upon a subjective feeling of just distribution. This finding is in line with results on the egocentric fairness bias (Tanaka, 1999), stating that especially just world believers (Rubin and Peplau, 1975) consider their own behavior as fairer than other people’s behavior. In relation to these findings, the perception of fairness might be considered biased by social demands (Blair, 2002), whereas actual fair behavior was counter-acted here by automaticity, i.e., number-space associations.

Several alternative explanations might also account for the observed general preference for the right player. In this regard, some limitations of the study have to be considered: Both the table coloring and the player characters were not counterbalanced and could have implied unidentified response tendencies^3^. The study sample was rather diverse regarding participants’ age, sex, and handedness, which likely increased the variance of number-space associations. Future studies should more closely examine these characteristics’ interactions with number-driven action decisions. By including the rummy card set, the stimuli used were, on one hand, of high external validity and allowed for instructing and investigating fair distribution behavior. On the other hand, the stimulus set by nature included two-digit and pictorial cards and thereby differs from previous studies. Nevertheless, we focused on single digits only in the mixed effects models analysis and thereby, the results of this analysis must be regarded exploratory and might underestimate the SNARC effect for the entire number range.

A closer look at single digits in the exploratory analysis pointed towards regular magnitude-space associations, but reversed parity-space associations. As such, automatic number magnitude processing emphasized a possible pre-existing preference bias by suggesting rightward (leftward) choices for high (low) value cards, resulting in higher scores. Given the full standard rummy card set, a regular MARC effect would have further emphasized responses favoring the right player. Placement preferences were increasingly identified in the literature, and the same is true for number-space associations. In a natural setting, it is likely that both types of bias affect choices, and our analysis confirms this view by the combination of identity-unspecific results (number of cards) and number specific results (scores and single-digit decision outcomes).

In conclusion, the results of our study support current views of actions as being influenced by language processing. During card distribution and while aiming at a fair and equal distribution, the participants’ choices were still affected by linguistic or conceptual features of actual rummy cards, namely digit parity and magnitude. A regular SNARC and a reversed MARC effect emerged and ultimately supported the overall preference of a right player avatar. The successful transfer of these effects to a more natural setting emphasizes the importance of further understanding the (neural) mechanisms behind indirectly and directly action-related linguistic and conceptual influences on number processing. Understanding these mechanisms will allow for identifying in which situations number associations can systematically bias behavior and, consequently, a better understanding will allow for countering these biases.

## AUTHOR CONTRIBUTIONS

PS and RP designed research; PS performed research; PS and RP analyzed data and wrote the paper.

### Conflict of Interest Statement

The authors declare that the research was conducted in the absence of any commercial or financial relationships that could be construed as a potential conflict of interest.

## Footnotes

1 As pointed out by a reviewer, individual variations of age, handedness or sex might play a role in marked decisions about numbers (see the discussion for an elaboration). However, also following the reviewer’s suggestion, fitting a model on right-handed female participants aged 30 or less did not substantially alter the results and only marginally improved the model fit.

2 Based on the comments of a reviewer, the model comparison was repeated using parametric bootstrapping with 1000 simulations, using the *PBmodcomp()* function of the R package pbkrtest (Halekoh and Højsgaard, 2014).

3 For effects of color on cognition, see Elliot and Maier (2014). We thank a reviewer for drawing our attention to this point.
